# Eravacycline as a Novel Option for Treating Multidrug-Resistant Gram-Negative and Gram-Positive Bacterial Infections: A Narrative Review

**DOI:** 10.3390/jcm15145617

**Published:** 2026-07-17

**Authors:** Aleksandra Złotowska, Julia Kacer, Zuzanna Dybko, Mateusz Dudek, Wiesława Duszyńska

**Affiliations:** 1 Student Scientific Association at the Department and Clinic of Anaesthesiology and Intensive Therapy, Faculty of Medicine, Wroclaw Medical University, L. Pasteura Street 1, 50-367 Wroclaw, Poland; aleksandra.zlotowska@student.umw.edu.pl (A.Z.); julia.kacer@student.umw.edu.pl (J.K.); zuzanna.dybko@student.umw.edu.pl (Z.D.); m.dudek@student.umw.edu.pl (M.D.); 2 Department and Clinic of Anaesthesiology and Intensive Therapy, Faculty of Medicine, Wroclaw Medical University, L. Pasteura Street 1, 50-367 Wroclaw, Poland

**Keywords:** eravacycline, MDR, Gram-negative bacteria, β-lactam antibiotics, antimicrobial resistance

## Abstract

**Background**: The alarming increase in the number of new bacterial defense mechanisms is exacerbating the problem of antibiotic resistance and poses a serious obstacle to the treatment of bacterial infections. Despite the approval of numerous new antibiotics by the Food and Drug Administration (FDA) and European Medicines Agency (EMA), mainly from the beta-lactam group since 2000, only some of them possess unique mechanisms of action and activity against multidrug-resistant Gram-negative bacilli (MDR GNB), including carbapenem resistance. Eravacycline (EVT), formerly known as TP-434, is a newly developed non-beta-lactam, fully synthetic tetracycline derivative, approved for the treatment of complicated intra-abdominal infections (cIAIs) in adults. **Results**: The analysis demonstrated significant efficacy of EVT across a wide range of in vitro and in vivo studies against MDR GNB, including carbapenem-resistant *Acinetobacter baumannii* (CRAB) and carbapenem-resistant Enterobacterales (CRE) and Gram-positive vancomycine-resistant pathogens. **Conclusions**: The broad spectrum of activity and favorable safety profile of EVT are well-documented in the treatment of critically ill patients. However, its efficacy against clinically relevant Gram-negative bacteria varies depending on specific species and resistance phenotypes, particularly among carbapenem-resistant and extensively drug-resistant (XDR) isolates.

## 1. Introduction

In recent years, the development of effective treatments for bacterial infections has become one of the main clinical challenges, due to the growing resistance of microorganisms to antibiotics [1]. According to the WHO, antimicrobial resistance is among the ten greatest global threats to public health [2]. Based on published data, it is predicted that, if left unchecked, drug-resistant infections could potentially cause over 10 million deaths annually by 2050 [3].

The prevalence of MDR-GNB varies depending on geographic location, healthcare facilities, and patient populations [4]. Higher rates are observed in intensive care units (ICUs) and long-term care facilities (LTCFs), which can act as reservoirs [5]. Rising incidence of infections caused by multidrug-resistant Gram-negative bacilli (MDR-GNB) leads to poor clinical outcomes, including increased mortality, longer hospital stays, and significantly higher healthcare costs [4]. Serious infections caused by these organisms, such as pneumonia, meningitis, surgical site infections, and bloodstream infections (BSIs), are of particular concern, especially in healthcare settings [6].

The pathogens of greatest concern include both GN and Gram-positive (GP) bacteria. These constitute the ESKAPE group of alert pathogens (E—*Enterococcus faecium* (*E. faecium*), S—*Staphylococcus aureus*, K—*Klebsiella pneumoniae* (*K. pneumoniae*), A—*Acinetobacter baumannii* (*A. baumannii*), P—*Pseudomonas aeruginosa* (*P. aeruginosa*), E—*Enterobacter* spp.), which pose the greatest threat in hospital-acquired infections [7]. The WHO’s new list of priority bacterial pathogens (BPPL) for 2024 is crucial for shaping global policy, research, development, and strategies in the fight against antimicrobial resistance (AMR). Carbapenem-resistant *A. baumannii* (CRAB), carbapenem-resistant Enterobacteriales (CRE), and third-generation cephalosporins-R, as well as rifampicin-resistant Mycobacterium tuberculosis, have been classified as critical priority pathogens, whilst carbapenem-resistant *P. aeruginosa* (CRPA), vancomycin-resistant (VR) Enterococcus faecium, methicillin-resistant *Staphylococcus aureus*, and others are classed as high-priority pathogens in the WHO BPPL 2024 [7,8].

The escalation of antibiotic resistance is due to inappropriate use, incorrect dosing, widespread development, overuse, and a shortage of targeted antimicrobial drugs [9]. Overuse of pharmaceuticals leads to bacteria developing mechanisms that reduce antibiotic susceptibility through mutations in chromosomal genes or genes constituting mobile genetic elements, as well as horizontal gene transfer [10]. This is a consequence of the natural phenomenon of selective pressure among microorganisms [11]. The alarming increase in new bacterial defense mechanisms exacerbates the problem of antibiotic resistance and poses a significant obstacle to the treatment of bacterial infections, even when antibiotics are used at clinically achievable concentrations [12].

The rise in antibiotic-resistant microorganisms poses a serious threat to the healthcare sector and requires innovative regimens and approaches [13]. Despite the advancement of antimicrobial treatment methods and the approval of numerous new antibiotics by the Food and Drug Administration (FDA) since 2000, only some of them possess unique mechanisms of action [13,14].

The significant financial and time burden associated with the development of new therapeutic agents has resulted in a decline in the development of new antibiotics, despite the clear need for them in medicine [15]. Alternative therapies, such as antigens, antibodies, probiotics, and vaccines, are most effective in clinical practice, primarily as adjuncts or preventative measures, as their efficacy and safety as stand-alone treatments have not been adequately validated [16].

The development of new antibiotic-based therapeutic interventions is crucial for controlling bacterial infections. The lack of effective antimicrobials can negatively impact countries at all levels of development [17]. With this in mind, we decided to conduct a narrative review examining the potential of eravacycline (EVT), highlighting its promising therapeutic benefits resulting from its high potency against MDR GNB and GP pathogens.

## 2. Materials and Methods

The information search was conducted in online databases containing large repositories of scientific research, namely MEDLINE, Scopus, Web of Science, and Google Scholar, which were systematically searched in May 2026. The search was limited to articles published in English and up to May 2026. The study also excluded articles for which full access could not be obtained. The search strategy was developed based on the research objective and included the following keywords: (“eravacycline” or “TP-434” or “Xerava”) AND (“multidrug-resistant” or “MDR” or “pan-drug resistant” or “PDR” or “Gram-negative” or “Gram-positive” or “carbapenem-resistant *Enterobacteriaceae*” or “CRE” or “Acinetobacter baumannii” or “CRAB” or “methicillin-resistant *Staphylococcus aureus*” or “MRSA” or “vancomycin-resistant Enterococci” or “VRE” or “complicated intra-abdominal infections” or “cIAI” or “mechanism of action” or “antimicrobial stewardship” or “clinical efficacy” or “safety profile”). Two independent reviewers conducted a comprehensive and objective literature review. In addition, the reference lists of relevant articles were manually reviewed, and a thorough search of the relevant literature was conducted to ensure the completeness of the results. The search procedure was repeated prior to the final analysis to include any newly published studies.

The data collection process was conducted manually through content analysis. The inclusion criteria (IC) that guide the preparation of this narrative literature review are explained as follows:

IC1: All original research articles, clinical trials, case series, and relevant medical reviews published in English.
IC2: Studies investigating the mechanism of action, pharmacological properties, in vitro/in vivo antimicrobial activity, clinical efficacy, or safety profile of EVT in the treatment of bacterial infections, particularly those caused by multidrug-resistant (MDR) Gram-negative and Gram-positive pathogens.
IC3: Studies employing observational, cohort, cross-sectional, interventional, translational, or microbiological experimental research designs (including in vitro susceptibility testing, minimum inhibitory concentration [MIC] assessments, and clinical outcome analyses).

The exclusion criteria (EC) were defined as follows:

EC1: Ineligible study design, including conference abstracts without full-text access, editorial letters, and unpublished data.
EC2: Ineligible clinical, microbiological, or pharmacological outcome measures, such as studies focusing purely on older-generation tetracyclines without comparative data for EVT, or studies lacking quantitative reporting of microbiological outcomes, clinical cure rates, or statistical analysis of the evaluated outcomes.
EC3: Ineligible study focus, including studies evaluating EVT solely for non-bacterial indications, or focusing exclusively on fully susceptible bacterial strains where multidrug resistance pathways are not addressed or relevant.

After removing duplicate records, titles and abstracts were independently screened by the first two authors (A.Z.; J.K.), and any discrepancies or final selections were subsequently reviewed and verified by a supervisor (W.D). Full texts of selected articles were then reviewed in detail, and studies were included or excluded based on predefined criteria. Only studies reporting the mechanism of action, antimicrobial activity, clinical efficacy, or safety profile of EVT in the treatment of multidrug-resistant bacterial infections were considered. Extracted data included: author(s); publication year; study design; target population and clinical indication (e.g., cIAI); evaluated bacterial pathogens and resistance patterns (e.g., MDR, CRE, CRAB); sample size (or number of bacterial isolates); methodology used (e.g., MIC testing, clinical trials, retrospective cohorts); dosing regimens; evaluated clinical or microbiological outcomes; and main findings.

In total, the literature review included 26 articles.

## 3. Chemical Structure, Mechanism of Action, and Registered Indications of Eravacycline

EVT, formerly known as TP-434, is a newly developed, fully synthetic tetracycline derivative approved for use in the United States on 27 August 2018, as well as in the European Union on 20 September 2018, for the treatment of complicated intra-abdominal infections (cIAIs) in adults [18]. It is important to note that, according to the 2026 Summary of Product Characteristics, EVT (marketed under the brand name Xerava) is indicated for the treatment of cIAIs in adults and adolescents aged 12 years and older weighing at least 50 kg [19]. EVT comprises a tetracyclic core skeleton and contains two unique modifications in the tetracyclic D-ring: a fluorine atom, which has been introduced at the C-7 position; and a pyrrolidinoacetam group, which has been added at the C-9 position [20]. Structural modifications of the tetracycline core through the introduction of additional substituents enable the overcoming of tetracycline-specific resistance mechanisms, such as reflux pumps and ribosomal hydrolysis [21]. Due to its unique structure, EVT demonstrated high stability in the presence of serine and metallo-β-lactamases in in vitro studies, as well as 2–4 times greater activity than tigecycline against CRE [22]. Crucial in clinical practice is the lack of cross-resistance with carbapenems, which allows for broad-spectrum treatment [23].

**Figure 1:**
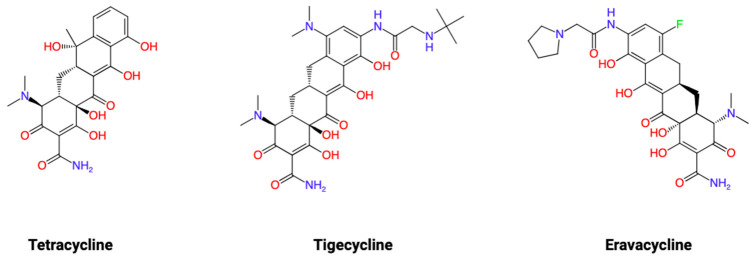
Chemical structures of tetracycline, tigecycline, and eravacycline. The structural progression shows the unmodified four-ring naphthacene core of tetracycline, the addition of a t-butylglycylamido group at the C-9 position in tigecycline (9-glycylcycline), and the dual modification of eravacycline (fluorocycline) featuring a fluorine atom at the C-7 position and a pyrrolidinoacetamido group at the C-9 position.

The mechanism of antibacterial action of EVT involves reversible binding to the bacterial 30S ribosomal subunit [24]. This prevents the incorporation of amino acid residues into elongating peptide chains, resulting in disruption of bacterial protein synthesis [20]. In an in vitro study by T. H. Grossman et al. [25], evaracycline’s affinity for ribosome binding was found to be ten-fold higher. Furthermore, the drug inhibited protein synthesis at four times lower concentrations than tetracycline [25]. EVT, like other tetracyclines, exhibits bacteriostatic activity [26]. Furthermore, it demonstrates bactericidal activity against certain strains of *A. baumannii*, *E. coli* and *K. pneumoniae* in vitro [27]. To highlight the pharmacological evolution and distinct clinical advantages within this antibiotic class, a comprehensive comparison of tetracycline, tigecycline, and eravacycline regarding their structures, dosing, and pharmacokinetic profiles is presented in Table 1. The structural evolution from the basic tetracycline core to the advanced modifications seen in modern analogs is illustrated in Figure 1, highlighting the key chemical changes that prevent modern resistance evasion.

## 4. The Spectrum of Activity of Eravacycline

EVT has a broad spectrum of activity against bacteria, such as GP, including methicillin-resistant *Staphylococcus aureus* (MRSA) and vancomycin-resistant (VR) *Enterococcus faecalis* (*E. faecalis*) and *Enterococcus faecium* (VRE); and GN aerobic, anaerobic—with exception of *P. aeruginosa*—and MDR GN strains, including extended-spectrum β-lactamase-producing *Enterobacteriaceae* and *Acinetobacter* [41]. It could also be effective against *Achromobacter* spp., *Stenotrophomonas maltophilia* (*S. maltophila*), *mycobacteria*, and even carbapenem-resistant *Enterobacterales* (CRE) [30].

### 4.1. Eravacycline Activity Against Gram-Negative Bacteria

EVT is used to treat patients with infections caused by carbapenemase-producing *Enterobacterales*. However, to clearly define its role in these infections, further clinical trials are necessary [42,43]. Comparing it with tigecycline, despite having similar mechanisms of action, it exhibits two- to four-fold higher activity against commonly encountered aerobic GNB. Its use should therefore be considered during the treatment of infections caused by carbapenem-resistant *Klebsiella pneumoniae* (CRKP) strains [44]. Clinical efficacy has been established for a range of *Enterobacterales*, including *Escherichia coli* (*E. coli*), *K. pneumoniae*, *Citrobacter freundii*, *Enterobacter cloacae*, and *Klebsiella oxytoca* [19]. The MIC_90_ (minimum inhibitory concentration) value for *E. coli* is 0.5 mg/L, whereas for *K. pneumoniae*, this is 2 mg/L [45]. The IGNITE4 study shows that in the treatment of cIAIs, EVT demonstrated non-inferiority (NI) in terms of clinical efficacy to meropenem. This is also consistent with the results of a previously published phase 3 study in which NI was proven relative to ertapenem [46]. EVT can also potentially be a potent antibiotic against non-fermenting GNB, especially *A. baumannii*. In in vitro studies, it shows greater efficacy than tigecycline, as its MIC_50/90_ values were two- to four-fold lower than those of tigecycline [47]. Its use is considered in cases of severe infections caused by this particular pathogen, as well as with carbapenem-resistant (CRAB) strains [48]. Unfortunately, its current use in this scope is limited, as standardized breakpoints are still lacking [48]. The spectrum of EVT activity also includes other non-fermenting GNB, such as *S. maltophilia* or *Achromobacter* spp. [49]. Furthermore, in vitro studies have shown that EVT may be a better choice for strains of *S. maltophilia* with exceptionally high resistance that are not susceptible to levofloxacin (LVFX), trimethoprim–sulfamethoxazole (TMP-SMZ), and minocycline [50].

### 4.2. Eravacycline Activity Against Gram-Positive Bacteria

In its activity against GP bacteria, EVT stands out for its effectiveness against MRSA, including strains also resistant to tetracycline. Studies reported the epidemiological cut-off (ECOFF) values against *S. aureus* are 0.25 mg/L [51]. In addition, it shows high efficacy in in vitro studies against VRE, namely *E. faecalis* (MIC_50/90_, 0.06/0.06 mg/L) and *E. faecium* (MIC_50/90_, 0.03/0.06 mg/L) [52]. Due to its broad range of activity against GP pathogens, EVT enables the treatment of cIAIs of mixed etiologies, effectively targeting both *Staphylococcus* and *Enterococcus* species. This reduces the necessity of using combination therapy [26,53]. Furthermore, its potency against MDR GP bacteria suggests a significant therapeutic potential in other conditions, such as skin and soft tissue or even respiratory tract infections [26,53].

### 4.3. Eravacycline Activity Against Anaerobes and Rare Pathogens

EVT exhibits activity against anaerobic bacteria, including *Bacteroides* spp. Clinical data also confirm its activity against other anaerobic pathogens, such as *Clostridium perfringens* and *Parabacteroides distasonis* [19]. This impacts its utility in the treatment of cIAIs and its significance in inhibiting the excessive use of carbapenems, which leads to the selection of strains resistant to them. Studies have also shown its minor effect on the intestinal microbiota, which reduces the risk of developing *Clostridioides difficile* infection (CDI) during antibiotic therapy. In one study, the frequency of this complication was noted in only one out of 50 patients undergoing therapy. This is of significant importance in patients after previous episodes of antibiotic-associated diarrhea [54]. In more recent preliminary studies, EVT has also been shown to be effective, both in vitro and in vivo, against rare pathogens like *Elizabethkingia anophelis*, even in regard to its MDR strains. This suggests the necessity of further research in this direction to standardize the therapy of infections with such etiology [55].

### 4.4. Gaps in the Spectrum and Limitations

Despite EVT’s great antibacterial capabilities, its limitations should be kept in mind. Some data indicate its activity against *Burkholderia cepacia* complex in in vitro studies, but other analyses show high MIC_90_ values (32 mg/L) for species, such as *Burkholderia cenocepacia*, suggesting their resistance [19]. It is also inactive in cases of infections caused by *P. aeruginosa*. Here, MIC_90_ values are also at the level of 32 mg/L [56]. Other studies have shown that in some strains of *K. pneumoniae*, overexpression of OqxAB and MacAB multidrug efflux pumps may occur, as well as the transcriptional regulator RamA, which most likely plays a part in the induction of resistance and heteroresistance to EVT [23,57]. Table 2 gathers the clinical MIC breakpoints and susceptibility categories for EVT against key bacterial pathogens.

For microorganisms such as *Acinetobacter* spp. or *S. maltophilia*, there is currently insufficient clinical evidence to establish standardized breakpoints, and susceptibility assessment is based on MIC distributions and pharmacokinetic data [19].

## 5. Eravacycline Dosage According to the Food and Drug Administration (FDA) and the European Medicines Agency (EMA)

### 5.1. Recommended Adult and Adolescents Aged 12 Years and Older Weighing at Least 50 kg Dosage

EVT is recommended at a dose of 1 mg/kg every 12 h by an intravenous infusion over 60 min. In cIAIs, treatment usually lasts 4 to 14 days, depending on the site and severity of infection and the patient’s clinical response [19].

### 5.2. Eravacycline Dosage for Patients with Hepatic Impairment

For patients with severe hepatic impairment (Child–Pugh C), EVT dosing should be reduced in frequency after the initial day of treatment, changing from every 12 h on day 1 to every 24 h from day 2 onward. No modification of the standard regimen is necessary in patients with mild-to-moderate hepatic impairment (Child–Pugh A or B) [19].

### 5.3. Eravacycline Dosage for Patients with Concomitant Use of a Strong Cytochrome P450 (CYP) Isoenzyme 3A Inducer

In patients with concurrent therapy with a strong CYP3A inducer, the dose should be increased to 1.5 mg/kg every 12 h [19].

### 5.4. Eravacycline Dosage for Patients with Renal Insufficiency and Hemodialysis

Dosage adjustment is not required in patients with renal impairment or in patients undergoing hemodialysis. Erythromycin should be administered regardless of the time of hemodialysis [19].

### 5.5. Eravacycline Dosage for Specific Pediatric Populations

The safety and efficacy of EVT in children less than 12 years of age or adolescents with body weight below 50 kg have not been established [19].

### 5.6. Eravacycline Dosage for Elderly Patients (≥65 Years Old)

No dose adjustment is required for elderly patients [19].

### 5.7. Special Clinical Situations

EVT, as a member of the tetracycline class, raises concerns regarding use during pregnancy, as this antibiotic class is associated with well-established developmental toxicity and is not recommended during pregnancy [58]. Clinical data on pregnant women are very limited and do not allow a reliable assessment of the risk of major congenital malformations or miscarriage [59]. Based on the known effects of tetracyclines, exposure during the second and third trimesters may cause discoloration of deciduous teeth and reversible inhibition of fetal bone growth [58]. Preclinical studies showed that EVT reached the fetal circulation and, when administered during organogenesis in rats and rabbits, was associated with post-implantation loss, lower fetal body weight, and delayed skeletal ossification [60].

There are no clinical data on the excretion of EVT into human breast milk. Animal studies have shown that the drug is present in milk, which may suggest a similar pattern in humans, although this has not been confirmed. Therefore, breastfeeding is not recommended during EVT treatment and for 4 days after the last dose.

## 6. Pharmacokinetics of Eravacycline

Preclinical and early clinical studies have consistently shown that EVT is characterized by extensive tissue distribution and favorable pharmacokinetic properties [61]. In a rabbit tissue distribution model, Petraitis et al. [62] demonstrated high concentrations of EVT in the lungs, bile, liver, urine, and renal cortex after repeated dosing, supporting broad tissue penetration. Following single intravenous doses of 1–10 mg/kg, mean AUC_0–168_ increased from 5.39 to 183.53 µg·h/mL in a dose-dependent manner (r = 0.97; *p* = 0.0001), although exposure appeared to increase non-linearly at doses ≥4 mg/kg. Notably, drug exposure in rabbits receiving 0.5–4 mg/kg was comparable to that observed in humans treated with 1 or 2 mg/kg every 12 h [62].

In randomized phase I studies conducted in healthy volunteers, intravenous EVT displayed broadly linear pharmacokinetics. Following single i.v. doses of 0.1–3 mg/kg, peak plasma concentrations were reached at the end of infusion, with a median Tmax of 0.5 h and a mean elimination half-life ranging from 12.7 to 25.6 h. With repeated dosing at 0.5–1.5 mg/kg for 10 days, a steady state was achieved within 5–7 days. At 1 mg/kg every 12 h, the accumulation ratio was approximately 45%; on day 10, this regimen produced an AUC_0–12_ of 6309 ng·h/mL and a Cmax of 1825 ng/mL. After a single dose, 13.4–18.8% of the administered drug was excreted unchanged in urine, suggesting that renal clearance plays only a limited role in overall elimination [34].

The routes of EVT elimination were characterized more precisely in a mass-balance study. After a single 60 mg intravenous dose of [14C]-EVT, 82.8% of the administered radioactivity was recovered, including 47.8% in feces and approximately 34% in urine, indicating that biliary–fecal excretion is the predominant elimination pathway. The volume of distribution was 217 L, further supporting extensive extravascular distribution [33].

Drug–drug interaction studies showed that itraconazole increased systemic exposure to EVT, raising AUC_0_–t by approximately 34% and prolonging the half-life from 14 to 19 h, while having only a minimal effect on Cmax. Clearance decreased concurrently from 3.79 to 2.57 mL/min/kg. In contrast, coadministration with rifampicin, a potent CYP3A4 inducer, had little effect on Cmax but reduced AUC_0_–t to 67.9% of the value observed in the absence of rifampicin and increased clearance by approximately 50%. Together, these findings indicate that strong CYP3A modulators can meaningfully alter EVT exposure [33].

Connors et al. [63] demonstrated substantial pulmonary penetration of EVT in healthy volunteers receiving 1.0 mg/kg every 12 h for seven doses. Mean concentrations in the epithelial lining fluid (ELF) were 0.70, 0.57, 0.34, and 0.25 µg/mL at 2, 4, 6, and 12 h, respectively, whereas the corresponding concentrations in alveolar macrophages were 8.25, 5.15, 1.77, and 1.42 µg/mL. These data translated into approximately six-fold higher exposure in ELF and 50-fold higher exposure in alveolar macrophages than in plasma, suggesting a possible use of EVT in lung infections [63].

These observations were subsequently reinforced by a population pharmacokinetic analysis showing a correlation between free EVT concentrations in plasma and ELF; the estimated ELF distribution coefficient was 8.26 [45].

According to data reported by Horn et al. [64], end-stage renal disease had only a minimal effect on EVT pharmacokinetics, with AUC_0_–inf 4% lower and Cmax 9% higher than in healthy participants. In contrast, hepatic impairment produced severity-dependent increases in exposure: AUC_0_–inf was elevated by 23%, 38%, and 110% and Cmax by 14%, 16%, and 20% in mild, moderate, and severe hepatic impairment, respectively [64]. The main pharmacokinetic and distribution parameters of the drug are detailed in Table 3.

## 7. The Place of Eravacycline in Current Guidelines for the Treatment of Infections Caused by Antibiotic-Resistant GNB and IAIs

According to our knowledge, at least four international guidelines on the treatment of infections caused by MDR GNB have been published in recent years under the auspices of professional associations, such as the Infectious Diseases Society of America (IDSA), the European Society of Clinical Microbiology and Infectious Diseases (ESCMID), the Spanish Society of Infectious Diseases and Clinical Microbiology (SEIMC), the Italian Society of Infectious and Tropical Diseases (SIMIT), and the French Society of Infectious Diseases (SPILF) [65,66,67,68]. Most of these organizations/societies list EVT as a therapeutic option for cIAIs caused by antibiotic-resistant GNB [65,66,67,68].

Although EVT is recommended in the guidelines for the treatment of MDR GNB, the following GPB, such as *Staphylococcus aureus*, the *Streptococcus anginosus* group, *E. faecalis*, *E. faecium*, and *Clostridium perfringens*—as well as GNB, such as *E. coli*, *K. pneumoniae*, *Enterobacter cloacae*, *Klebsiella oxytoca*, *Bacteroides* species, *Citrobacter freundii*, and *Parabacteroides distasonis*; and anaerobic pathogens—have also demonstrated clinical susceptibility to EVT [65,66,67,68].

Experts who commented on the NICE review of the scientific evidence regarding the use of EVT for the treatment of cIAIs, published in 2022, emphasized that, in practice, EVT is most commonly prescribed to patients allergic to penicillin or in situations where standard intravenous antibiotics are unsuitable or have proven ineffective. This is consistent with NICE guidelines on the rational use of antibiotics [69].

According to the ESCMID guidelines by M. Paul et al. [67] on the treatment of MDR GNB, EVT demonstrates potential in vitro activity against CRAB, ESBL, non-CP CRE, KPC-CRE, OXA-48-CREn and MBL-CRE. As EVT shows no in vitro/in vivo activity against Pseudomonas, the same guidelines do not recommend EVT for non-MBL CRPA [67]. The ESCMID guidelines include recommendations on the use of EVT for the treatment of multidrug-resistant GNB. With regard to the use of EVT in the treatment of intravascular infections caused by GN strains resistant to third-generation cephalosporins, EVT was compared with ertapenem (39 patients with ESBL-producing Enterobacterales) [67]. The authors found non-inferiority of EVT compared with carbapenem because clinical or microbiological outcomes were similar between groups [67].

The same guidelines emphasize that, in the case of ceftazidime with avibactam, there is moderate certainty regarding efficacy in the treatment of IAIs caused by ESBL-producing Enterobacteriales, whereas for EVT and ceftolozane with tazobactam, the certainty is very low [67]. In regard to IAIs caused by CRE, it should be noted that EVT exhibits twice the in vitro activity of tigecycline against GNB. Furthermore, the studies that led to the drug’s approval did not include any patients with infection caused by CRE; consequently, there is no evidence to support the efficacy of EVT in the treatment of abdominal infections caused by these pathogens [67]. In terms of EVT’s efficacy against CRAB, its MIC is two to eight times lower than that of tigecycline against CRAB. It has been evaluated in two RCTs in comparison with ertapenem and meropenem for the treatment of cIAIs [67]. Despite the drug’s potential activity in vitro, there are no data available on its clinical efficacy against CRAB infections [67].

Following analysis of the IDSA guidelines by P.D. Tamm et al. [68], EVT is recommended for the treatment of IAIs caused by GN, such as CRE and KPC (with the exception of bloodstream and urinary tract infections), similar to tigecycline, as well as for MBL-producing CRE (including NDM) and OXA-48, with the exception of bloodstream and urinary tract infections [68].

The fourth updated version of the IDSA guidelines focuses on infections caused by Enterobacterales-producing extended-spectrum β-lactamases (ESBL-E), Enterobacterales-producing AmpC β-lactamases (AmpC-E), CRE, *P. aeruginosa* with difficult-to-treat resistance (DTR *P. aeruginosa*), CRAB, and *S. maltophilia*. The authors of this document emphasized that clinical data on the treatment of CRAB with EVT are limited [68].

In an observational study involving 93 patients with CRAB pneumonia, the use of EVT was associated with a longer duration of mechanical ventilation (11 vs. 7 days) and higher 30-day mortality (33% vs. 15%) compared with alternative treatment regimens. All four patients with CRAB BSIs who received EVT died [68]. Given the limited clinical data supporting the efficacy of EVT, the panel suggests restricting its use to situations where other drugs are ineffective, not tolerated, or unavailable. In the same document, EVT is also not recommended for the treatment of infections caused by *S. maltophilia* and *P. aeruginosa* [68].

In the executive summary of the SEIMC consensus document on the diagnosis and treatment of infections caused by carbapenem-resistant (CR) GNB, authored by Pintado V et al. [70], the recommendations regarding the use of EVT are very limited. These guidelines focused on pathogens, their resistance, and antibiotic dosing rather than on the site of infection, and suggested treatment for infections caused by CRE, carbapenem-resistant *Pseudomonas aeruginosa*, CRAB, and *S. maltophilia*. With regard to EVT, the guidelines mention this only once, stating that there is insufficient evidence to recommend EVT for the treatment of infections caused by multidrug-resistant *A. baumannii* [70].

The Italian (SIMIT) and French (SPILF) consensus documents, which propose a ‘practical approach’ to key situations not covered by the ESCMID and IDSA guidelines, are additional tools for selecting optimal antibiotics in cases of severe infections caused by MDR-GNB; however, EVT is not listed as a drug used to treat IAIs caused by these pathogens [65].

According to the Polish “Guidelines for the treatment of infections caused by CPE *Enterobacteriaceae*” by W. Hryniewicz et al. [71], EVT is indicated in several situations where complicated IAIs are diagnosed. Firstly, EVT is recommended for the treatment of infections caused by CPE, similar to ceftazidime/avibactam, meropenem/vaborbactam, and cefiderocol. Secondary indications for EVT are infections caused by *Enterobacteriaceae*-producing MBLs (VIM, IMP, NDM), similar to cefiderocol and ceftazidime/avibactam + aztreonam. An additional condition for which EVT may be used is for infections caused by OXA-48 *Enterobacteriaceae*. In all cases, susceptibility to EVT must be confirmed microbiologically [71].

Regarding the Brazilian guidelines published by Zavascky et al. [72] in 2025 on the treatment of infections caused by difficult to treat, these were defined by the World Health Organization (WHO) as pathogens of critical importance and high priority, including CRE, CRAB, *P. aeruginosa* (CRPA), and ESBL- and AmpC-producing Enterobacterales, as well as *S. maltophilia* and *Burkholderia cepacia*. Treatment recommendations are organized by pathogen and site of infection, including respiratory tract, skin and soft tissue, blood, abdominal, and urinary tract infections. The authors did not enumerate EVT in these guidelines and concluded that, despite the approval of several new agents by the EMA and FDA, many of these antimicrobial agents remain inaccessible in Brazil due to limited market interest and delays in obtaining national regulatory approval. Furthermore, the panel points out that the few new antimicrobial agents available on the Brazilian market represent first-line treatment options for most CR-GN bacilli. However, these novel agents are not available in the majority of public hospitals [72].

The working group on “Guidelines Recommendations for Evidence-based Antimicrobial use in Taiwan” (GREAT), whose draft was developed and published by Cheng Len Sy et al. [73] in 2022 under the title “Recommendations and guidelines for the treatment of infections due to multidrug resistant organisms”, covered infections caused by CRAB, CRPA, CRE, and VRE. The authors of these guidelines listed EVT for the treatment of complicated IAIs caused by CRE alongside other options, such as ceftazidime/avibactam + metronidazole, imipenem/cilastatin/relebactama, tigecycline, or combinations of colistin + tigecycline or meropenem [73].

In accordance with the guidelines published by Sartelli M. et al. [74], issued by the Italian Council for the management of IAIs and the optimization of antimicrobial use, EVT is strongly recommended in a number of clinical situations. The authors recommend the use of EVT in empirical therapy for complicated appendicitis, complicated cholecystitis, acute cholangitis, acute diffuse peritonitis, intra-abdominal abscess, non-traumatic small bowel perforation, and perforated gastric and duodenal ulcers (in critically ill patients with inadequate/delayed control of the infection focus and a high risk of infections caused by ESBL-producing Enterobacteriales or in cases of septic shock), in the same way as the use of appropriate carbapenems. In patients with postoperative peritonitis without intestinal colonization by MDR strains and in immunocompetent patients, the use of EVT is recommended, as is meropenem, doripenem, and imipenem/cilastatin. In patients with postoperative peritonitis and suspected MDR etiology based on epidemiological data and/or data on intestinal colonization and/or risk factors for MDR infection, the use of imipenem/cilastatin–relabactam, meropenem/vaborbactam, or ceftazidime/avibactam + linezolid or teicoplanin is recommended. In patients at high risk of intra-abdominal candidiasis, liposomal amphotericin B or echinocandins should be added [74].

In contrast, for severe acute pancreatitis, the guidelines state that empirical antibiotic therapy should be reserved strictly for treating infected necrosis. In this specific clinical scenario, EVT is recommended for immunocompetent patients without MDR colonization, serving as an alternative to meropenem, doripenem, or imipenem/cilastatin. However, if an MDR etiology is suspected—due to epidemiological data, gut colonization, or specific risk factors—the authors advise using imipenem/cilastatin/relabactam, meropenem/vaborbactam, or ceftazidime/avibactam combined with linezolid or teicoplanin (plus antifungal therapy for patients at high risk of intra-abdominal candidiasis). Additionally, the document highlights that EVT is indicated as a crucial alternative to beta-lactams for patients with documented beta-lactam allergies, both in severe acute pancreatitis and in various forms of respiratory tract infections [74].

## 8. In Vitro Evaluation of Eravacycline Activity

X. Yang et al. [47] observed that EVT exhibits greater in vitro antibacterial efficacy against *A. baumannii* compared with tigecycline. The study analyzed 2144 *A. baumannii* isolates (2021–2024) from a Chinese tertiary referral hospital for resistance trends. It was found that the MIC50/90 values for EVT were 2–4 times lower compared to tigecycline. Subinhibitory concentrations of EVT induced increased resistance among the tested isolates to EVT, tigecycline, minocycline, and polymyxin B, whilst PAβN reversed these effects. In 97.06% of strains, tigecycline’s MIC was ≥4 µg/mL, and the induced EVT MIC values exceeded achievable alveolar concentrations. Resistance to EVT correlated with tigecycline; however, the use of combination therapy with polymyxin B did not show significant synergistic activity. The study suggests the need for caution regarding resistance caused by the rapid increase in the expression of efflux pump genes, highlighting the need for the prudent use of EVT in clinical practice to limit the development of resistance [47].

Y. Zhong et al. [75] evaluated the activity of EVT against clinical CRAB isolates from Singapore and investigated the genetic profiles of these isolates, taking into account new potential mechanisms of resistance to EVT through whole-genome sequencing (WGS) analysis. EVT exhibited strong in vitro activity against CRAB, with an MIC90 value of 2 µg/mL. WGS analysis revealed that 300 CRAB isolates belonged to 23 STs, with ST2 being the dominant population, frequently harboring blaOXA-type endogenous β-lactamases and tetracycline-class efflux pumps (tet(B)). Furthermore, the emergence of resistance to EVT (elevated MIC values and MIC > MIC90) was detected. Concurrently, genetic analysis of isolates with high MIC values enabled the identification of novel mutations in the resistance–nodulation–division (RND) efflux pump genes (adeABC, adeFGH, and adeIJK) and their regulatory elements (adeS/R and adeL/N). The study highlights that EVT is a promising therapeutic option for the treatment of CRAB infections in Singapore; however, the emergence of resistance mediated by new genetic mutations necessitates continuous surveillance and further research [75].

A study by M. Zhou et al. [76] reported that EVT exhibits promising in vitro activity against CRKP isolates from pediatric patients. The susceptibility rates of 74 CRKP strains to TGC and EVT were 100% and 87.8%, respectively. For both antibiotics (TGC and EVT), MIC values ranged from 0.125 to 2 μg/mL; the MIC50 was 0.25 μg/mL, whilst the MIC90 reached 1 μg/mL. EVT consistently had lower or equal MIC90 values compared with TGC for typical STs, with the exception of ST11, where the MIC50 value for EVT was also lower or equal. EVT demonstrated significant in vitro activity against pediatric CRKP isolates collected over a 6-year period, indicating potential clinical utility in this demographic group [76].

B. D. Johnston et al. [77] determined MIC values in broth microdilutions for cefiderocol, ceftazidime–avibactam, and EVT, as well as 11 comparator drugs, against 343 clinical carbapenem-resistant E. coli isolates (CR). The collection comprised 203 isolates from the USA (2002–2017) and 141 isolates from 17 countries in Europe, Latin America, and the Asia–Pacific region (2003–2017). Cefiderocol, ceftazidime–avibactam, and EVT showed the highest susceptibility rates (82% to 98%) to tigecycline (99%) [77].

In the study by Y. S. Huang et al. [78], 40 clinical CRKP isolates were evaluated—half were KPC while the other half were MBL. Significant differences in ceftazidime/avibactam MIC values were observed between KPC and MBL isolates. A checkerboard analysis revealed synergy between tigecycline (35%) or EVT (40%) and polymyxin B, regardless of the type of isolates, maintaining a synergy rate of 70.6% even for polymyxin B-resistant isolates. Combinations with ceftazidime/avibactam showed a significantly lower level of synergy (tigecycline 5%, EVT 10%). Among the MBL CRKP isolates, only one showed synergy with these combinations, and elimination time analysis did not reveal any synergistic activity. The combination of EVT and polymyxin B showed the most promising synergy [78].

In a study by N. Słabisz et al. [79], 60 NDM-producing *K. pneumoniae* strains obtained from various patients hospitalized at the 4th Military Hospital in Wrocław between 2019 and 2022 were evaluated. Among the antibiotics tested, the highest susceptibility (100%) was observed for cefiderocol, EVT, and tigecycline [79].

A study by S. Hu et al. [55] confirmed the high antimicrobial efficacy of EVT and omadacycline against *E. anophelis*. The study included 197 clinical isolates. MIC testing revealed strong in vitro activity against clinical isolates, with MIC values of 0.06 mg/L and 0.125 mg/L, respectively. The data obtained confirm the validity of using EVT and omadacycline in the in vitro eradication of multidrug-resistant *E. anophelis* and indicate the need for further clinical trials [55].

## 9. Clinical Trials Conducted Before the Registration of Eravacycline

Pre-registration studies have shown that EVT has promising activity against MDR GNB.

In a phase 2, double-blind, randomized trial conducted by J. Solomkin et al. [80], the efficacy and safety of two EVT dosing regimens were evaluated in comparison with ertapenem in adult patients hospitalized with cIAIs. Patients with confirmed cIAIs requiring surgical or percutaneous intervention and antimicrobial treatment were randomized (2:2:1) into groups receiving EVT at a dose of 1.5 mg/kg body weight every 24 h, EVT at a dose of 1.0 mg/kg every 12 h, or ertapenem at a dose of 1 g every 24 h for a period of 4 to 14 days. In the ME population, the clinical success rate at the follow-up visit (TOC) was 92.9% (39/42) in the group receiving EVT at a dose of 1.5 mg/kg every 24 h, 100% (41/41) in the group receiving EVT at a dose of 1.0 mg/kg every 12 h, and 92.3% (24/26) in the group receiving ertapenem. The study reported comparable efficacy between the two regimens and ertapenem in patients with cIAI [80].

J. Solomkin et al. [81] conduct a randomized, double-blind, multicenter phase 3 trial—IGNITE1—evaluating the efficacy and safety of EVT compared with ertapenem in patients with cIAI requiring surgical or percutaneous intervention. A total of 541 patients were enrolled and randomly assigned to the EVT or ertapenem groups. Study participants received ertapenem at a dose of 1.0 g every 24 h for at least four 24 h dosing cycles. A small difference in the clinical cure rate was observed at the cure assessment visit between the two groups (86.8% for EVT vs. 87.6% for ertapenem). The difference in the clinical cure rate between the groups was −0.80% (95% CI: −7.1% to 5.5%), which fell within the pre-specified equivalence margin, confirming the statistical equivalence of EVT and ertapenem [81].

In a prospective, randomized, double-blind phase 3 trial—IGNITE4—by J. S. Solomkin et al. [46], EVT demonstrated comparable efficacy to meropenem in the treatment of cIAIs at the primary endpoint (90.8% vs. 91.2%). In the analysis of secondary endpoints, the clinical cure rate was assessed in the modified ITT population (92.4% vs. 91.6%) and in the clinically evaluated population (96.9% vs. 96.1%). In both cases, the results obtained were comparable between the drugs under investigation. The cure rate in patients with ESBL-producing *Enterobacteriaceae* was 87.5% (14/16) and 84.6% (11/13) in the EVT and meropenem groups, respectively [46].

## 10. Real-World Effectiveness and Retrospective Analyses of Eravacycline in Post-Registration Studies

### 10.1. Pathogen-Specific Efficacy and Clinical Outcomes

Y. Li et al. [82] conducted a retrospective, multicenter study evaluating the clinical efficacy of EVT and its correlation with MICs in infections caused by *A. baumannii* or *K. pneumoniae*. The analysis included 1796 adult patients with infections caused by *A. baumannii* or *K. pneumoniae* in China. The overall susceptibility rate to EVT was 96.0% (1027/1070), while 98% of CR isolates were susceptible to EVT. Further analysis showed that EVT had a MIC90 four times lower than tigecycline against both pathogens. Upon completion of the treatment course, therapeutic success (microbiological eradication or resolution of clinical symptoms) was recorded in 82.6% of patients in the cohort, and microbiological eradication was observed in 76.1%. By day 30, the infection had been cured in 83.57% of cases. No correlation was found between different EVT treatment regimens (monotherapy or combination therapy) and clinical outcomes after 30 days. In the multivariate analysis, a positive association was noted between elevated C-reactive protein (CRP) levels during treatment, BSI, sepsis, specific clinical interventions, and treatment failure [82].

J. Chen et al. [83] carried out a single-center, retrospective study involving lung transplant recipients with CRAB infections who received EVT for more than 72 h. The median duration of EVT was 10 days, with the majority of patients receiving combination therapy. A 28-day survival rate of 83.3% was reported, whilst secondary endpoints showed a 100% 14-day survival rate and a 37.5% clinical failure rate. The results obtained indicate significant clinical efficacy of EVT in lung transplant recipients with CRAB infection, providing valuable evidence and clinical experience regarding its use in post-organ transplant populations [83].

S. R. V. Helden et al. [84], in a study evaluating the efficacy of EVT in the treatment of enterococcal infections, included 100 adult patients who received EVT for ≥72 h. *E. faecalis* and *E. faecium* were present in 44% and 58% of patients, respectively, and VRE accounted for 49% of all isolates. The 30-day mortality rate was 17%, and infection-related mortality within 30 days was 12%. Re-hospitalization and biological recurrence within 30 days were observed in 14% and 4% of cases, respectively [84].

S. Giuliano et al. [85] analyzed two cases of BSIs caused by *S. maltophilia* in immunocompromised patients. Both patients received EVT as part of combination therapy following microbiological identification of the pathogen. EVT was administered in both treatment regimens at a dose of 1 mg/kg every 12 h. Blood cultures cleared within 48 h in both cases. One of the patients died during follow-up; however, microbiological cures of *S. maltophilia* BSIs was achieved in both cases. These results suggest a potential role for EVT in the treatment of *S. maltophilia* BSIs when standard options are limited [85].

### 10.2. Eravacycline in the Management of Bacterial Pneumonia

In a multicenter retrospective study by Z. Luo et al. [86] involving 113 adult patients, the clinical efficacy of EVT was assessed in the treatment of various infections in patients hospitalized in respiratory wards in China. After 30 days of treatment, the clinical efficacy rate was 87.6%. Microbiological eradication was achieved in 85.8% of cases, whilst the survival rate was 85.0%. Strong EVT activity against MDR pathogens was confirmed, particularly *A. baumannii* (*n* = 51) and *K. pneumoniae* (*n* = 27). No positive correlation was observed between monotherapy (*n* = 70) and combination therapy regimens (*n* = 43). The study demonstrated the high efficacy and tolerability of EVT in the treatment of patients in respiratory wards, particularly those caused by MDR GN pathogens [86].

Q. Guo et al. [87] retrospectively compared the efficacy of EVT and tigecycline in the treatment of CRAB-induced pneumonia in 65 ICU patients. Patients in the EVT group had a longer duration of antibiotic therapy prior to administration (19.79 ± 9.97 days vs. 14.25 ± 7.98 days; *p* < 0.05) and a longer median hospital stay [27 (19.50, 39.00) days] compared with the tigecycline group [19 (14.00, 29.00) days]. Mortality within 30 days was lower in the EVT group than in the tigecycline group (15.2% compared with 25.0%). Furthermore, no deaths were recorded among patients treated with EVT who consulted an infectious disease specialist. The study demonstrated that EVT is no less effective than tigecycline in the treatment of CRAB-induced pneumonia whilst offering a favorable safety profile. Prompt consultation with an infectious disease specialist and timely initiation of EVT therapy following a sentinel culture are key factors influencing improved clinical outcomes [87].

### 10.3. Multicenter Real-World Evidence and Clinical Experience

In a multicenter, retrospective study, A. Hobbs et al. [88] demonstrated the potential versatility of EVT, characterized by its broad spectrum of activity, as well as its flexibility in use in patients with renal impairment or antibiotic allergies. The study included 66 patients. EVT monotherapy was used in 62.1% of the subjects. The mean duration of treatment was 13.1 ± 9.9 days. In the majority of patients (68.2%), EVT was used off-label, including 34.8% for lung infections and 28.8% for skin and soft tissue infections. Among the isolated microorganisms, 50% were CR GN pathogens and 48% were VR GP pathogens in vitro. Clinical improvement was observed in 95.5% of cases, whilst complete resolution of the infection was observed in 86.4% of patients. The lack of clinical improvement in three patients was associated with inadequate control of the IAIs. At the same time, six patients, in whom the infection did not resolve completely, died from causes unrelated to the infection during hospitalization [88].

N. Van Hise et al. [89], in a retrospective observational cohort study, analyzed the efficacy of EVT in the treatment of IAIs, pneumonia, and diabetic foot infections in critically ill patients. EVT was administered at a dose of 5 mg/kg every 24 h to 50 patients. All patients completed treatment outside the hospital. Clinical resolution of the infection was achieved in 94% of cases. Clinical failure was recorded in 6% of patients. IAI (36%), pneumonia (18%), diabetic foot (12%), spontaneous bacterial peritonitis (8%), and empyema (8%) were the most common conditions among patients. In just under half of the infections, more than one pathogen was isolated, and resistant strains were common. Clinical efficacy in real-world conditions was comparable to the results of clinical trials (94%) [89].

S. Alosaimy et al. [90] evaluated the use of EVT in 35 patients for the treatment of various infections. Primary sources of infection included IAIs (34%, 12/35), followed by the respiratory tract (29%, 10/35), bones/joints (14%, 5/35), and skin/soft tissues (9%, 3/35). Positive blood cultures accounted for 34% (12/35) of all clinical isolates, with the main source being intra-abdominal (42%, 5/12). Ultimately, remission was achieved in all patients with positive blood cultures (100%, 12/12). However, in 58% (7/12), recovery occurred before the start of EVT treatment. The most frequently isolated pathogens included *K. pneumoniae*, *E. faecium* and *A. baumannii*. The 30-day survival rate was 74%. No recurrence within 30 days and resolution of infection symptoms were observed in 91% and 57% of cases, respectively. The study authors emphasize that EVT yielded favorable outcomes in terms of survival, protection against recurrence, and resolution of symptoms despite the presence of risk factors for clinical failure in the majority of patients [90].

In a multicenter, retrospective study by K. Patino et al. [91], the efficacy and safety of EVT were evaluated in the treatment of MDR infections within an academic healthcare system in Atlanta, Georgia. The study included 48 subjects who received EVT for infections caused by VRE (31.3%), *S. maltophilia* (29.2%), and CRE (20.8%). The median duration of treatment with EVT was 12.6 days. Treatment failure was recorded in 39.6% of cases, whilst 60.4% of patients survived without recurrence. Multivariate analysis did not identify any significant factors contributing to treatment failure due to a lack of association [91].

S. Giuliano et al. [92] conducted a retrospective study evaluating the real-world use of EVT in 13 patients hospitalized for complicated infections at a single center. The clinical cure rate was 69.2%, and the mortality rate was 38.5%. The deaths of the patients were clearly associated with resistance mechanisms, such as VIM-producing *K. pneumoniae* and *Enterobacter cloacae*, and inadequate control of the source of infection. The authors of the study emphasized the significant efficacy of EVT in MDR infections [92].

In a retrospective study conducted by A. J. Kunz Coyne et al. [93], clinical success was reported in 75.7% of patients treated with EVT. Among these, survival and the absence of microbiological recurrence within 30 days of completing EVT treatment were observed in 94.7% and 94.5% of patients, respectively, whilst 84.1% showed clinical improvement within 96 h of starting EVT treatment. Most patients who died within 30 days of completing EVT treatment (5.3%) had positive sputum (40.9%) or blood (31.8%) culture results. The most commonly isolated pathogens were *Enterococcus* spp. (31.8%), *K. pneumoniae* (18.2%), and *S. maltophilia* (18.2%). The study results suggest that EVT is mainly used as a consolidation therapy in monobacterial infections of various etiologies. However, the broad spectrum of activity of EVT also makes it an effective therapeutic option in infections caused by MDR pathogens [93].

A study by J. Wang et al. [94] evaluated the efficacy and safety of EVT in Chinese patients with hematologic diseases. Of the 796 patients enrolled in the study, the majority had hematologic diseases (94.6%) and had recently undergone chemotherapy or radiation therapy (80.2%). The most common infection was pneumonia (57.4%), and the most common type of specimen for pathogen isolation was sputum (47.2%). Among the 481 patients who had microbiological test results, *Klebsiella pneumoniae* (30.5%) and *Acinetobacter baumannii* (17.4%) were the most common pathogens. The mean time to resolution of fever was 3.2 ± 2.1 days. The overall clinical response rate was 88.8%, with response rates of 84.0% for bloodstream infections and 87.5% for pulmonary infections. The microbiological response rate at the end of treatment was 90.7%. EVT demonstrated high susceptibility rates against *A. baumannii* (95.8%), *K. pneumoniae* (94.3%), and *Staphylococcus aureus* (100.0%). Adverse events were reported in only 2.5% of patients. Subgroup analysis showed that pulmonary disease (*p* = 0.006), sepsis (*p* = 0.003), and a duration of treatment with eravacycline at a dose of 1 mg/kg every 12 h of ≤7 days (*p* < 0.001) were significantly associated with a poorer clinical response rate at the end of treatment [94].

K. Oberreiter et al. [95] conducted a retrospective study involving 78 patients from Austria and Udine to assess the real-world efficacy of EVT in various sites of infection and against various pathogens using descriptive statistics. EVT was most commonly used for intra-abdominal infections (44.9%), followed by pneumonia (12.8%) and infections of unknown origin (7.7%). The most common pathogen was Escherichia coli, including ESBL-producing strains (24.4%), followed by *Enterococcus* spp. (12.8%) and *Klebsiella pneumoniae* (12.8%). Clinical cure was achieved in 65% of patients, while microbiological cure was observed in 46%; containment of the infection was achieved in 48.7%, and 16.7% of patients died within 30 days. A total of 48% of patients required intensive care [95].

### 10.4. Clinical Impact of Variable Dosing Regimens on Safety and Efficacy

N. M. McCourt and colleagues [96] assessed the efficacy and safety of alternative EVT dosing strategies in a single-center, retrospective cohort study. Two different EVT dosing regimens were used to treat various non-mycobacterial infections susceptible to EVT in 133 patients. Similar rates of clinical efficacy were observed in both groups (54% for once-daily dosing vs. 46% for twice-daily dosing). However, once-daily dosing was associated with lower mortality (4% vs. 26%) and a higher rate of adverse events [96].

### 10.5. Emerging Evidence and Preliminary Clinical Data

In a retrospective case series, A. Carr et al. [97] evaluated the efficacy of EVT in clinical practice in 23 patients who received at least two doses of the drug. EVT was administered to the subjects for an off-label indication or for a microorganism in 91% of cases. Microorganisms, such as *A. xylosoxidans*, *S. maltophilia*, CRAB, and *K. pneumoniae*, were isolated from blood cultures. A total of 48% of respondents had EVT susceptibility ranging from 0.06 to 4 µg/mL, including two MICs ≥ 32 µg/mL for *S. maltophilia*. The median duration of EVT was 8 (3–30) days. Clinical efficacy was achieved in 57% of patients. Clinical failure was recorded in nine cases. Among these, treatment was switched to an alternative in 14% of patients, and in 10% of cases, there was no improvement in symptoms. The study authors emphasize the significant difference between treatment outcomes in clinical trials and in real-world conditions [97]. The results are summarized in Table 4.

## 11. Safety, Tolerability, and Adverse Event Profile of Eravacycline

The favorable safety profile and tolerability of EVT in the treatment of infections have been confirmed in numerous clinical trials [87]. In the IGNITE4 trial by J. Solomkin et al. [46], its efficacy was observed in five out of five patients infected with *A. baumannii* with cIAI [46]. Although EVT was evaluated for oral administration, clinical trials failed to demonstrate its efficacy regarding the co-primary endpoints of clinical cure and microbiological success within the microbiological intent-to-treat population at the test-of-cure visit. Among the leading adverse effects are gastrointestinal events, nausea (6.5%) and vomiting (3.8%) predominated, followed by diarrhea [30,49]. In addition, other frequently observed side effects include hepatotoxicity, pancreatitis, hypofibrinogenemia, thrombophlebitis, prolonged coagulation profile, migratory phlebitis, and allergic reactions at the injection site [30,49,98,99,100]. Concurrently, adverse events were observed more frequently in patients treated with other antibiotics, including ertapenem, compared to EVT [81]. Studies also noted a significant advantage in the tolerability of EVT compared to that of tigecycline [46]. Furthermore, EVT is used in patients who are allergic to beta-lactam antibiotics and cannot tolerate carbapenems [101]. Compared to antibiotics of a similar class, EVT is characterized by a relatively low incidence of side effects [46]. As it belongs to the tetracycline group, typical complications such as tooth discoloration, enamel hypoplasia, skeletal growth retardation, and *Clostridioides difficile*-associated diarrhea should be taken into account [101,102]. In a study evaluating the efficacy of EVT in treating infections in hematological patients conducted by J. Wang et al. [94], a clinical response rate of 88.8% and a microbiological response rate of 90.7% were observed [94]. According to clinical studies, patients with impaired renal function may also benefit from treatment with EVT, as evidenced by the antibiotic’s lack of nephrotoxicity [88]. Despite the proven efficacy of EVT in the adult population, it has not been included in pediatric indications [103]. However, in vitro studies on isolated strains of CRKP have shown its significant therapeutic efficacy and thus its potential for off-label use [76]. An unexpected finding from the case report by M. Melchio et al. [104] was the absence of a need for dose adjustment of EVT in a patient with moderate hepatic impairment (Child–Pugh class B) [104].

## 12. Resistance to Eravacyline

Regarding antibiotic resistance, it is more frequently observed in GNB, especially those from the *Enterobacteriaceae* family and in *P. aeruginosa* and *A. baumannii* [105]. However, in this context, the broad spectrum of antibacterial activity of EVT exhibits significant gaps due to intrinsic resistance; in particular, *P. aeruginosa* and members of the tribe Proteae (including *Proteus* spp., *Providencia* spp., and *Morganella morganii*) are inherently resistant to this drug due to constitutional, chromosomally encoded multidrug efflux pumps [23,106]. A marked variation in the susceptibility profile to EVT is observed among bacteria of the order Enterobacterales, with higher resistance in *K. pneumoniae* and *E. cloacae*, *Morganellaceae*, and significantly lower in *E. coli* [23]. The introduction of EVT was intended to overcome classical mechanisms of resistance to tetracyclines, which is reflected in its MIC values that are similar to, or slightly more favorable than, those of tigecycline [107]. The tet(K) and tet(L) genes encoding efflux pumps, as well as the tet(M) genes encoding ribosome protection proteins, play a key role in tetracycline resistance [108]. The accumulated data demonstrate minimal inhibition of EVT activity by specific efflux pumps, ribosome protection proteins, and enzymatic inactivation [56]. The inclusion of efflux pump inhibitors in therapy may increase treatment efficacy due to a significant reduction in EVT’s MIC values in resistant subpopulations and a reduction in the development of heteroresistance to this antibiotic among CRAB isolates [109]. This role is fulfilled by CCCP (m-chlorophenylhydrazone carbonyl cyanide) [110]. A significant correlation was found between *A. baumannii* resistance to EVT and the presence of the ISAba1 insertion sequence within the *adeS* gene [111]. This sequence determines the overexpression of the adeABC efflux pump system [112]. The mechanism of action of the adeABC efflux pump involves pumping the antibiotic out of the cell, thereby reducing its efficacy [113]. It is particularly noteworthy that strains carrying the ISAba1 insertion mutation within another gene—*adeN*—which were resistant to tigecycline in the study by S. Gerson et al. [114], retained sensitivity to EVT [114]. In a study by F. Zhang et al. [115], by analyzing the resistance of *S. aureus* strains, it was shown that *MSSA* strains were characterized by higher MIC values and a greater prevalence of heteroresistance compared to *MRSA* isolates [115]. This resistance is also associated with a mutation within the 30S subunit of the ribosome, which results in impaired EVT binding [116]. The optimal approach for carbapenem-resistant strains of *E. coli* and *K. pneumoniae* appears to be combination therapy with EVT and polymyxin B [117]. This combination maintained therapeutic efficacy even against resistant strains whilst exhibiting a synergistic effect of 70.6% [78]. Furthermore, promising results have been achieved with the combination of EVT and ceftazidime–avibactam or aztreonam/avibactam in the case of a clinical isolate of K. pneumoniae-producing NDM-1 (Kp17). In addition, evaluation of the heteroresistance phenomenon in *E. faecalis* to EVTs showed that it is caused by overexpression of the gene encoding the BMP family ABC transporter substrate-binding protein (RS00630) [44,118]. The prevalence of EVT-resistant strains within the order Enterobacterales ranges from 0.9% to 41.9%; specifically, resistance in *E. coli* varies from 0.9% to 9.6% among consecutive isolates and from 0% to 29% among selected isolates, whereas in *K. pneumoniae*, it reaches 10% to 41.9%. Resistance in *A. baumannii* was estimated at 43.4%, although this rate was substantially lower in the CRAB and CSAB subgroups at 2.4% and 1.3%, respectively [119].

## 13. Study Limitations

Most studies suggest that EVT is highly effective in treating infections caused by MDR GNB. However, the limited sample size and significant heterogeneity of the study populations prevent drawing clear clinical conclusions. This heterogeneity resulted from differences in patient characteristics (age, gender, comorbidities), different study protocols (measurement methods, duration of follow-up), and inconsistencies in outcome classification. Further studies involving larger patient cohorts and applying uniform criteria for assessing efficacy and safety are necessary to precisely assess EVT’s therapeutic potential.

## 14. Conclusions

EVT represents a valuable addition to the antimicrobial armamentarium against MDR pathogens, offering a potent carbapenem-sparing therapeutic alternative. Its broad-spectrum activity and favorable safety profile are well-documented; however, its efficacy against clinically significant GNB exhibits variability across specific species and resistance phenotypes, particularly among CR and extensively drug-resistant (XDR) isolates. Accordingly, clinical deployment should be informed by local epidemiological data and antimicrobial susceptibility testing. While current evidence supports EVT use in severe and polymicrobial-complicated IAIs, further high-quality clinical trials are warranted to validate its efficacy in off-label indications and to optimize its integration into antimicrobial stewardship frameworks.

## Figures and Tables

**Table 1:** Comparative analysis of the structural, microbiological, and pharmacokinetic characteristics of tetracycline, tigecycline, and EVT.

Feature	Tetracycline	Tigecycline	Eravacycline	Key Literature Sources
Chemical Class/Generation	First generation tetracycline	9-Glycylcycline (third generation)	Fluorocycline (fourth generation)	Zhanel et al. [28]; Chopra et al. [29]
Chemical Structure Modification	Basic four-ring naphthacene carboxamide core, without modifications at C-7 or C-9.	Addition of a t-butylglycylamido group at the C-9 position of the minocycline core.	Fluorine atom modification at the C-7 position and a pyrrolidinoacetamido group at the C-9 position.	Scott [24]; Zhanel et al. [28]
Spectrum of Activity	Broad spectrum originally; currently highly limited due to widespread resistance. Retains utility for atypical pathogens (*Chlamydia*, *Mycoplasma*).	Expanded broad spectrum: MRSA, VRE, ESBL-producing Enterobacterales, *Acinetobacter baumannii*, and anaerobes. Lacks activity against *Pseudomonas aeruginosa*.	Enhanced broad spectrum: highly potent against CRE, CRAB, MRSA, VRE, and multidrug-resistant anaerobes. Lacks activity against *P. aeruginosa*.	Zhanel [28]; M. Albanell-Fernández et al. [30]; M. L. Townsend et al. [31]
Key Resistance Mechanisms	Highly susceptible to both efflux pumps (e.g., Tet(A)-(E)) and ribosomal protection proteins (e.g., Tet(M)).	Overcomes classic efflux and ribosomal protection. Susceptible to upregulated chromosomal multidrug efflux pumps (MexXY-OprM, adeABC).	Engineered to completely evade classic ribosomal protection and efflux pumps. Retains high potency against many upgraded Enterobacterales efflux systems.	Scott et al. [24]; Chopra & Roberts [29]; Ruzin et al. [32];
Pharmacokinetics (Vd & Half-Life)	Vd: 0.14–1.6 L/kg (significantly lower tissue distribution compared to glycylcyclines/fluorocyclines).	Vd: significantly larger (>10 L/kg) than traditional tetracyclines (0.14–1.6 L/kg).	Vd: ~3.1 L/kg (217 L, supporting extensive extravascular distribution)	Zhanel et al. [28]; Newman et al. [33]; Newman et al. [34]; Klein et al. [35]
	Serum half-life (t1/2): 6–12 h. Highly dependent on renal excretion.	Mean half-life (t1/2): ~36 h in humans. Less than 15% is excreted unchanged in the urine (biliary/fecal excretion predominant).	Mean elimination half-life (t1/2): ranges from 12.7 to 25.6 h in healthy volunteers. Predominantly cleared via biliary–fecal excretion.	
Dosing & Administration	Total 1 g daily, divided as 500 mg twice daily (b.i.d.) or 250 mg four times daily (q.i.d.). For severe infections, dosage may be increased to 500 mg four times daily (q.i.d.).	Loading dose of 100 mg intravenously (i.v.), followed by 50 mg i.v. every 12 h (q12h).	A total of 1 mg/kg intravenously every 12 h.	Ref. [19]; Shutter et al. [36]; Greer et al. [37]
Available Formulations	Oral (capsules, tablets), topical, intramuscular, and intravenous (i.v.).	Intravenous (i.v.) powder for reconstitution.	Intravenous (i.v.) powder for reconstitution.	Shutter et al. [36]; Jitkova et al. [38]; Ref. [19]
FDA/EMA Approved Indications	Acne vulgaris, respiratory tract infections, chlamydia, rickettsial infections, and alternative for syphilis.	Complicated skin and skin structure infections (cSSSIs), complicated intra-abdominal infections (cIAIs), and CABP.	Complicated intra-abdominal infections (cIAIs).	Chopra et al. [29]; Townsend et al. [31]; Ref. [19]
Main Clinical Advantages	Low cost, long-standing history of use, and oral availability for mild/chronic conditions.	Evades early tetracycline resistance mechanisms; broad empiric coverage including anaerobes and MRSA without renal adjustments.	Significantly greater in vitro potency (lower MICs) against CRE and CRAB than tigecycline; better GI tolerability.	Sapadin et al. [39]; Yaghoubi et al. [40]; Chen et al. [22]

**Table 2:** Clinical MIC breakpoints * for EVT, according to EUCAST (European Committee on Antimicrobial Susceptibility Testing) guidelines [19].

Pathogen	Susceptible ** (S≤) [mg/L]	Resistant *** (R>) [mg/L]
*Escherichia coli*	0.5	0.5
*Staphylococcus aureus*	0.25	0.25
*Enterococcus* spp.	0.125	0.125
*Streptococcus viridans* group	0.125	0.125

* MIC breakpoints were established for the treatment of complicated cIAIs at standard intravenous dosing (1 mg/kg body weight every 12 h). ** Category S: Strains characterized by a level of susceptibility that ensures a high probability of therapeutic success. *** Category R: Strains for which there is a high probability of therapeutic failure.

**Table 3:** Overview of the key pharmacokinetic (PK) parameters and tissue distribution profiles of eravacycline.

Parameter/Property	Key Pharmacokinetic Findings	Authors of the Study
Absorption and Peak Plasma Levels (Cmax/Tmax)	Broadly linear pharmacokinetics following single i.v. doses (0.1–3 mg/kg). Peak plasma concentrations achieved at the end of infusion (median Tmax = 0.5 h). At 1 mg/kg q12h (day 10), Cmax reaches 1825 ng/mL and AUC_0–12_ of 6309 ng·h/mL	Newman et al. 2018 [34]
Volume of Distribution (Vd) and Tissue Distribution	Extensive extravascular and tissue distribution; volume of distribution is high at 217 L. Animal models show high concentrations in lungs, bile, liver, urine, and renal cortex.	McCarthy 2019 [61]; Petraitis et al. 2018 [62]; Newman et al. 2019 [33]
Pulmonary Penetration (ELF and Alveolar Macrophages)	Substantial lung penetration. Mean concentrations at 1.0 mg/kg q12h: ELF (0.25–0.70 µg/mL; ~6-fold higher than plasma) and alveolar macrophages (1.42–8.25 µg/mL; ~50-fold higher than plasma). Population PK analysis shows an ELF distribution coefficient of 8.26.	Connors et al. 2014 [63]; Ji et al. 2025 [45]
Elimination Half-Life (t1/2) and Accumulation	Mean elimination half-life ranges from 12.7 to 25.6 h in healthy volunteers. Steady state achieved within 5–7 days with repeated dosing (0.5–1.5 mg/kg for 10 days). Accumulation ratio is ~45% at 1 mg/kg q12h.	Newman et al. 2018 [34]
Excretion and Elimination Pathways	Predominantly biliary–fecal excretion. Mass-balance study [14C]-EVT shows 82.8% total radioactivity recovery: 47.8% in feces and ~34% in urine. Only 13.4–18.8% is excreted unchanged in urine (renal clearance plays a limited role).	Newman et al. 2018 [34]; Newman et al. 2019 [33]
Hepatic Impairment Impact	Severity-dependent increases in exposure. AUC_0_–inf increases by 23% (mild), 38% (moderate), and 110% (severe impairment). Cmax increases by 14% (mild), 16% (moderate), and 20% (severe).	Horn et al. 2017 [64]
Renal Impairment Impact	End-stage renal disease (ESRD) has only a minimal effect on PK parameters (AUC_0_–inf decreased by 4%, Cmax increased by 9%). No major dosage adjustments needed based purely on renal function.	Horn et al. 2017 [64]
Drug–Drug Interactions (CYP3A4 Modulators)	Strong CYP3A modulators meaningfully alter exposure. Co-administration with itraconazole (inhibitor) increases AUC_0_–t by ~34% and extends half-life from 14 to 19 h. Co-administration with rifampicin (potent inducer) reduces AUC_0_–t to 67.9% and increases clearance by ~50%.	Newman et al. 2019 [33]

**Table 4:** Summary of in vitro, in vivo, and clinical efficacy of EVT.

	Study Type	Objective	Type of Infection	Sample Size	EVT Monotherapy	EVT Combination Therapy	Comparator	Pathogen	Key Findings	Author of the Study
1	In vitro experimental study	Evaluation of the impact of sub-MIC EVT exposure on CRAB and analysis of the resistance mechanisms.	CRAB infections	2144 isolates (primary analysis); 43 strains (specific testing)	Yes	Not applicable	Tigecycline (efficacy comparison) and baseline strains vs. induced resistant strain	*A. baumannii*	Sub-MIC EVT exposure induces resistance via up-regulation of *adeB* and *adeS* efflux pumps. Resistance is largely mutation independent (88.37% of strains), though mutations stabilize the resistance.	X. Yang et al. [47]
2	In vitro susceptibility study and whole-genome sequencing (WGS) analysis	Evaluation of the activity of EVT against CRAB clinical isolates from Singapore and investigation of genetic profiles and novel potential EVT resistance mechanisms.	CRAB infections	300 clinical CRAB isolates	Yes	Not applicable	Other antibiotics (susceptibility testing comparison)	*A. baumannii* (predominantly ST2)	EVT showed strong activity (MIC90 of 2 µg/mL). Resistance is linked to novel mutations in RND efflux pump genes (*adeABC*, *adeFGH*, *adeIJK*) and their regulators.	Y. Zhong et al. [75]
3	In vitro susceptibility and comparative analysis study	Assessment of the in vitro activities of EVT and tigecycline against CRKP isolates obtained from pediatric patients in China.	CRKP infections	74 pediatric CRKP strains	Yes	Not applicable	Tigecycline	*K. pneumoniae* (CRKP)	EVT susceptibility was 87.8% (MIC90: 1 µg/mL). Susceptibility was primarily determined by carbapenemase type, being lowest in *blaKPC-2* (64.3%) and highest in *blaNDM-1* (97.4%). Strains from children > 3 years old showed significantly lower susceptibility (46.7%) compared to neonates/toddlers (100%).	M. Zhou et al. [76]
4	In vitro susceptibility study (broth microdilution) and molecular characterization	Evaluation of the activity of EVT, CFDC, and CZA against CR clinical *E. coli* isolates in relation to bacterial characteristics and region.	Carbapenem-resistant *Escherichia coli* (CR *E. coli*) infections, including extraintestinal infections	344 clinical isolates	Yes	Not applicable	11 comparators, including tigecycline, cefiderocol, and ceftazidime–avibactam	*E. coli* (including ST131, ST405, and ST648)	EVT exhibited high susceptibility (82–98%), ranking just below tigecycline (99%). Susceptibility was significantly higher in isolates carrying the CTX-M beta-lactamase. Regional variation was noted, with lower susceptibility in the South and Southeast U.S.	B. D. Johnston et al. [77]
5	In vitro synergy study (checkerboard and time–kill analysis)	Evaluation of the in vitro efficacy of selected antibiotic combinations against CRKP infections, specifically those producing KPC and MBL.	Carbapenem-resistant *Klebsiella pneumoniae* (CRKP) infections	40 clinical isolates (20 KPC-producing, 20 MBL-producing)	Yes	Yes (EVT + Polymyxin-B and EVT + ceftazidime/avibactam)	Tigecycline, polymyxin-B, and ceftazidime/avibactam	*K. pneumoniae* (CRKP)	EVT + polymyxin-B showed 40% synergy in checkerboard analysis and the most promising synergy in time–kill analysis. EVT + ceftazidime/avibactam showed significantly lower synergy (10%). Synergy with polymyxin-B remained high (70.6%) even against polymyxin-resistant strains.	Y. S. Huang et al. [78]
6	In vitro drug susceptibility and antibiotic combination assessment	Assessment of the in vitro drug susceptibility of NDM-producing *K. pneumoniae* strains to antibiotics recommended by IDSA and ESCMID.	NDM-producing *K. pneumoniae* infections (hospitalized patients)	60 NDM-producing *K. pneumoniae* strains	Yes	Yes (specifically evaluated ceftazidime/avibactam + aztreonam via “strip stacking”)	Cefiderocol, tigecycline, fosfomycin, and ceftazidime/avibactam + aztreonam	*K. pneumoniae* (New Delhi metallo-β-lactamase [NDM] producers)	EVT demonstrated 100% sensitivity (EUCAST interpretation). Cefiderocol and tigecycline also showed 100% sensitivity. The combination of ceftazidime/avibactam + aztreonam was highlighted as the best first-line therapeutic option due to 100% in vitro sensitivity and safety.	N. Słabisz et al. [79]
7	Preclinical study (in vitro + in vivo);	Evaluation of EVT and omadacycline efficacy against *E. anophelis* in vitro and in vivo.	*E. anophelis* bacteremia (animal model)	197 clinical *E. anophelis* isolates; *Galleria mellonella* larvae; BALB/c murine bacteremia model (*n* = 12 per group)	Yes	Not applicable	Omadacycline; SPSS control	MDR *E.anophelis*	EVT showed potent in vitro and in vivo activity against *E. anophelis*. In the murine bacteremia model, EVT achieved complete bacterial eradication in hepatic, splenic, and pulmonary tissues and reduced inflammatory markers.	S. Hu et al. [55]
8	Phase 2, randomized, double-blind, active-control study	Evaluation of efficacy, safety, and pharmacokinetics of two EVT dose regimens (1.5 mg/kg q24h IV and 1.0 mg/kg q12h IV) versus ertapenem in hospitalized adults with cIAIs.	cIAIs	143 (53 received EVT 1.5 mg/kg q24h, 56 EVT 1.0 mg/kg q12h, 30 ertapenem)	Yes	Not applicable	Ertapenem	GN aerobes (67.9%), GP aerobes (23.6%), anaerobes (8.5%); *E. coli* (60.3%), *E. faecalis* (6.7%), *B. fragilis* (5.0%)	In the microbiologically evaluable population, TOC clinical cure was 92.9% for EVT 1.5 mg/kg, 100% for EVT 1.0 mg/kg, and 92.3% for ertapenem; both EVT regimens were as efficacious as comparator and well tolerated.	J. Solomkin et al. [80]
9	Phase 3, randomized, double-blind, double-dummy, multicenter study (IGNITE1)	Assessment of efficacy and safety of EVT compared with ertapenem in adults hospitalized with cIAIs.	cIAIs	541 (EVT *n* = 270, ertapenem *n* = 271)	Yes	No	Ertapenem	GN and GP aerobic and anaerobic bacteria	In the microbiological intent-to-treat population, TOC clinical cure was 86.8% with EVT and 87.6% with ertapenem meeting the prespecified non-inferiority margin. Both drugs were well tolerated.	J. Solomkin et al. [81]
10	Phase 3, randomized, double-blind, double-dummy, multicenter study (IGNITE4)	Demonstration of the non-inferiority of EVT versus meropenem based on clinical cure at test-of-cure in the micro-ITT population.	cIAIs	500 (EVT *n* = 250, meropenem *n* = 250)	Yes	No	Meropenem	GP and GN aerobes and anaerobes; *Enterobacteriaceae*; ESBL-producing; CRE	EVT was non-inferior to meropenem in the primary endpoint: 90.8% vs. 91.2%. In ESBL-producing *Enterobacteriaceae*, cure was 87.5% with EVT vs. 84.6% with meropenem.	J. Solomkin et al. [46]
11	Retrospective, multicenter study	Assessment of the real-world effectiveness of EVT and the relationship between clinical effectiveness and MIC values.	Pneumonia, BSIs, IAIs, UTIs, CNS infections caused by *A. baumannii* or *K. pneumoniae*	1796	Yes (75.0% of patients)	Yes (*n* = 449); mainly polymyxins (17.1%), cefoperazone–sulbactam (2.4%), imipenem (1.3%)	Not applicable	*A. baumannii* (*n* = 1214), *K. pneumoniae* (*n* = 582); including CR strains	EOT treatment success was 82.6%, microbiological eradication 76.1%, and day-30 infection cure 83.57%; AEs: 2.28%; no significant difference between monotherapy and combination therapy; predictors of failure: elevated CRP, BSIs, and sepsis.	Y. Li et al. [82]
12	Retrospective, single- center study	Evaluation of the clinical effectiveness of EVT for the treatment of CRAB infections in lung transplant recipients.	CRAB infections in lung transplant recipients	24 lung transplant recipients	Yes (12.5%)	Yes (87.5%); with 1 antibiotic (45.8%), 2 antibiotics (33.3%), 3 antibiotics (8.3%); agents: cefoperazone–sulbactam, polymyxins, carbapenems	Not applicable	CRAB	Clinical resolution: 62.5%; clinical failure: 37.5%. At 14-day survival: 100%; 28-day survival: 83.3%; 28-day mortality: 16.7%; predictors of failure: older age, ILD, septic shock, CRRT.	J. Chen et al. [83]
13	Retrospective subgroup analysis of real-world efficacy and safety	Assessment of the real-world efficacy and safety of EVT for the treatment of *E. faecalis* or *E. faecium* infections.	*Enterococcus* infections (primarily IAIs, skin/soft tissue, and osteomyelitis)	100 adult patients	Yes, 100 patients (received EVT for ≥72 h)	Not applicable	Baseline infection signs/symptoms (pre-treatment vs. post-treatment)	*E. faecalis* (44%) and *E. faecium* (58%), including 49% VRE isolates	Clinical cure was achieved in 77% of patients. The 30-day mortality was 17%, and the 30-day readmission rate was 14%. EVT was well-tolerated, with only 2% of patients requiring discontinuation due to adverse events.	S. R. V. Helden et al. [84]
14	Clinical case series (review of two cases)	Investigation of the potential role of EVT in treating *S. maltophilia* BSI when standard therapeutic options are limited.	BSIs in immunocompromised patients (AML and cholangiocarcinoma)	2 patients	No	Yes (1 mg/kg every 12 h as part of a regimen)	Not applicable	*S. maltophilia*	Blood cultures cleared within 48 h in both cases. While one patient died from fungal complications, the *S. maltophilia* BSI was microbiologically controlled in both patients, suggesting EVT as a potential option for MDR pathogens.	S. Giuliano et al. [85]
15	Multicenter, retrospective, real-world clinical study	Evaluation of the real-world clinical effectiveness and safety of EVT in treating various infections in patients hospitalized in respiratory departments.	Various infections (primarily respiratory/pulmonary) caused by MDR pathogens	113 adult patients	Yes, 70 patients	Yes, 43 patients	EVT-based combination therapy (e.g., with colistin, meropenem, tigecycline, or other antibiotics based on susceptibility testing)	MDR pathogens, specifically *A. baumannii* (*n* = 51) and *K. pneumoniae* (*n* = 27)	EVT showed 87.6% clinical efficacy and 85.8% microbiological eradication. The 30-day survival rate was 85.0%. Notably, there was no statistically significant difference in outcomes between monotherapy and combination therapy.	Z. Luo et al. [86]
16	Retrospective clinical study	Comparison of the clinical efficacy and adverse reactions of EVT and tigecycline as target therapies for CRAB pneumonia in ICU patients.	CRAB pneumonia (often involving co-infections with *Aspergillus* and *K. pneumoniae*)	65 ICU patients	Yes, 33 patients (EVT group)	Not applicable	Tigecycline	CRAB	EVT demonstrated non-inferior efficacy of tigecycline. The 30-day mortality was lower in the EVT group (15.2% vs. 25.0%). Notably, zero mortality was observed in EVT patients who received infectious disease specialist consultations.	Q. Guo et al. [87]
17	Retrospective, multicenter clinical evaluation	Characterization of early clinical experience, utilization patterns, and outcomes of EVT in a real-world setting.	Various (68.2% off-label), including pulmonary (34.8%), skin/soft tissue (28.8%), and IAIs	66 patients	Yes, 41 patients (62.1% of cases)	Yes, 25 patients (37.9% of cases)	EVT-based combination therapy (e.g., meropenem, cefepime, vancomycin, piperacillin/tazobactam, ceftolozane/tazobactam, fluoroquinolones, aminoglycosides)	Diverse MDR pathogens: 50% of GN (CR) and 48% of GP (VR).	In total, 95.5% clinical improvement and 86.4% full infection resolution. The drug was well-tolerated with only 4.5% adverse events (nausea/vomiting). No cases of *C. difficile* were reported during therapy.	A. Hobbs et al. [88]
18	Retrospective observational cohort study	Evaluation of the efficacy and safety of EVT in a range of infections such as intra-abdominal infections, pneumonia, and diabetic foot infections in seriously ill patients.	Various: IAI (36%), pneumonia (18%), diabetic foot (12%), spontaneous bacterial peritonitis (8%), and empyema (8%)	50 consecutive patients	Yes, 50 patients (evaluated as the primary treatment)	Not applicable	Not applicable	MDR species (including ESBL or KPC-producing isolates); 50% of cases were polymicrobial	Overall, 94% clinical resolution rate (47/50 patients). The drug was highly effective despite 88% of patients having ≥2 comorbidities. EVT was well-tolerated with only two cases of nausea and one case of *C. difficile* within 30 days post-therapy.	N. Van Hise et al. [89]
19	Multicenter, retrospective observational study	The clinical and safety outcomes among patients treated with EVT in the real-world setting.	IAIs, respiratory tract infections	35 adult patients, most patients were male	Yes	Yes (51%) cefepime (13%), meropenem (13%), and polymyxin B (13%)	EVT monotherapy versus combination therapy	*K. pneumoniae, E. faecium*, and *A. baumannii.*	High 30-day survival and absence of 30-day recurrence in the majority of patients. Demonstrated robust efficacy in patients at high risk for clinical failure.	S. Alosaimy et al. [90]
20	Multicenter, retrospective study	To evaluate the real-world efficacy and safety of EVT for the treatment of MDR infections within an academic-based health system in Atlanta, Georgia.	Blood, respiratory tract, or wound(s)	48 adult patients, most patients were female	Yes	Yes (77%) vancomycin, micafungin, or meropenem	EVT monotherapy versus combination therapy	VRE, *S. maltophilia*, and CRE	Valuable agent for infections with limited therapeutic options due to broad-spectrum activity. Favorable safety profile, with a lower rate of adverse effects (4.2%) compared to other agents. High tolerability, making it a preferred alternative in critically ill or MDR-infected patients.	K. Patino et al. [91]
21	Retrospective study	Real-world EVT use in hospitalized patients treated for complicated infections.	IAIs	13 adult patients	Yes	Yes	Not applicable	CRE and *A. baumannii*	The significant efficacy of EVT in multidrug-resistant (MDR) infections.	S. Giuliano et al. [92]
22	Retrospective, cohort, observational study	Assessment of efficacy (clinical/microbiological) and adverse effects of EVT therapy among hospitalized patients in the U.S.	Respiratory tract (24.8%), wound(s) (20.9%), blood	416 adult patients	Yes	Yes (meropenem (17.5%) or amikacin (8.5%)	Not applicable	*Enterobacterales* spp. (24.4%, CR), *Enterococci* spp. (49.0%, VR), and *Acinetobacter* spp. (47.4%, CR)	Broad-spectrum activity against MDR GN and GP pathogens with low MIC90 values. Potential therapeutic option for challenging infections, including CRE and *Stenotrophomonas maltophilia*. Effective alternative in complex clinical scenarios where standard MDR therapies are limited. Noteworthy efficacy against carbapenem-resistant Acinetobacter spp., despite lack of official FDA indication.	A. J. Kunz Coyne et al. [93]
23	Single-center, retrospective cohort study	Comparative analysis of safety and efficacy between two regimens: 1.5 mg/kg q24h (once daily) vs. 1 mg/kg q12h (twice daily).	IAIs (48%)	133 adult, hospitalized patients	Yes	Not applicable	Not applicable	*E. faecium* (24.3%) and *E. coli* (27.8%)	Equivalent clinical success rates demonstrated between the ERVD (1.5 mg/kg q24h) and ERVBID (1 mg/kg q12h) regimens. No statistically significant differences in adverse events observed between the two dosing groups. Study findings support a consistent safety profile for both once-daily and twice-daily EVT administration.	N. M. McCourt et al. [96]
24	Retrospective study	The utility of EVT in clinical practice.	Respiratory (44%), cIAIs	23 adult patients	Yes	Yes (83%)	Not applicable	*A. xylosoxidans, S. maltophilia*, CRAB, and *K. pneumoniae*	Significant discrepancies between initial real-world use and clinical trials regarding illness severity and infection types. Real-world application involves more critically ill patients and a broader range of infections than investigated in phase 3 trials. Clinical outcomes in practice differ from trial results, reflecting the complexity of treating high-acuity, MDR-infected populations.	A. Carr et al. [97]
25	Multicenter, retrospective, real-world study	Evaluation of the effectiveness and safety of EVT in Chinese hematology patients.	Severe bacterial infections (most common: pneumonia [57.4%], pulmonary infections, bloodstream infections, and sepsis)	796 patients (including 481 patients with microbiological examination results)	Yes, 402 patients (50.5%)	Yes, 394 patients (49.5%)	Not applicable	*K. pneumoniae* (30.5%), *A. baumannii* (17.4%), and *Staphylococcus aureus*	The overall clinical response rate was 88.8% (84.0% in bloodstream infections and 87.5% in pulmonary infections). The microbiological response rate at the end of treatment was 90.7%. EVT exhibited high susceptibility rates: 100.0% for *S. aureus*, 95.8% for *A. baumannii*, and 94.3% for *K. pneumoniae*. The mean time to defervescence was 3.2 ± 2.1 days.	J. Wang et al. [94]
26	Retrospective, descriptive, real-world study	Evaluation of the real-world efficacy of eravacycline in various infection sites and pathogens.	Various infection sites, most commonly intra-abdominal infections (44.9%), pneumonia (12.8%), and infections of unknown origin (7.7%)	78 patients (48% requiring intensive care)	Yes, 9 patients (11.5%)	In total, 30 patients (38.5%) received a *Pseudomonas*-effective regimen. Most common combination partners were fosfomycin (18%), meropenem (15.4%), and trimethoprim/sulfamethoxazole (13%). Documentation was missing for 13 patients (16.7%).	Not applicable	*E. coli* (including ESBL producers; 24.4%), *Enterococcus* spp. (12.8%), and *K. pneumoniae* (12.8%)	Clinical cure was achieved in 65% of the overall population and 73.6% of the fixed-dose cohort (200 or 300 mg daily). Microbiological cure was documented in 46% of patients, and source control was attained in 48.7%. The overall 30-day mortality rate was 16.7%. Two deaths in the high-dose cohort were linked to a lack of source control and severe underlying conditions.	K. Oberreiter et al. [95]

*A. baumannii*—*Acinetobacter baumannii*; *A. xylosoxidans*—*Achromobacter xylosoxidans*; *B. fragilis*—*Bacteroides fragilis*; BSI—bloodstream infection; cIAI—complicated intra-abdominal infection; CNS—central nervous system; CR—carbapenem-resistant; CRAB—carbapenem-resistant *Acinetobacter baumannii*; CRE—carbapenem-resistant Enterobacterales; CRKP—carbapenem-resistant *Klebsiella pneumoniae*; CRRT—continuous renal replacement therapy; *E. anophelis*—*Elizabethkingia anophelis*; *E. coli*—*Escherichia coli*; *E. faecalis*—*Enterococcus faecalis*; *E. faecium*—*Enterococcus faecium*; EVT—eravacycline; FDA—Food and Drug Administration; GN—Gram-negative; GP—Gram-positive; IAI—intra-abdominal infection; ILD—interstitial lung disease; ICU—intensive care unit; *K. pneumoniae*—*Klebsiella pneumoniae*; MDR—multidrug-resistant; MIC—minimum inhibitory concentration; UTI—urinary tract infection; *S. maltophilia*—*Stenotrophomonas maltophilia*; VR—vancomycin-resistant; VRE—vancomycin-resistant *Enterococcus*.

## Data Availability

No new data were created or analyzed in this study.

## Abbreviations

*A. baumannii*	*Acinetobacter baumannii*
*B. fragilis*	*Bacteroides fragilis*
BSI	Bloodstream infection
cIAI	Complicated intra-abdominal infection
CNS	Central nervous system
CR	Carbapenem-resistant
CRAB	Carbapenem-resistant *Acinetobacter baumannii*
CRE	Carbapenem-resistant Enterobacterales
CRKP	Carbapenem-resistant *Klebsiella pneumoniae*
*E. coli*	*Escherichia coli*
*E. faecalis*	*Enterococcus faecalis*
*E. faecium*	*Enterococcus faecium*
ESBL-E	Extended-spectrum β-lactamases
ESCMID	European Society of Clinical Microbiology and Infectious Diseases
EVT	Eravacycline
EMA	European Medicine Agency
FDA	Food and Drug Administration
GN	Gram-negative
GNB	Gram-negative bacteria
GP	Gram-positive
IAI	Intra-abdominal infection
IDSA	Infectious Diseases Society of America
ICU	Intensive care unit
*K. pneumoniae*	*Klebsiella pneumoniae*
LVFX	Levofloxacin
MDR	Multidrug-resistant
MIC	Minimum inhibitory concentration
*P. aeruginosa*	*Pseudomonas aeruginosa*
*S. maltophilia*	*Stenotrophomonas maltophilia*
SEIMC	Spanish Society of Infectious Diseases and Clinical Microbiology
SIMIT	Italian Society of Infectious and Tropical Diseases
SPILF	French Society of Infectious Diseases
TMP-SMZ	Trimethoprim–sulfamethoxazole
*VR*	Vancomycin-resistant
VRE	Vancomycin-resistant *Enterococcus*
